# Cognitive Distortions as Barriers to Seeking Smoking Cessation Treatment: A Comparative Study

**DOI:** 10.3390/jcm13133974

**Published:** 2024-07-07

**Authors:** Selim Arpacıoğlu, Erkal Erzincan, Mine Ergelen, Beyza Arpacıoğlu, Salih Cihat Paltun, Murat Yalçın, Rabia Bilici

**Affiliations:** 1 Psychiatry Department, Faculty of Medicine, Altınbaş University, Istanbul 34147, Turkey; selim.arpacioglu@altinbas.edu.tr; 2 Psychology Department, Faculty of Economics Administrative and Social Sciences, Gelişim University, Istanbul 34310, Turkey; erkal.erzincan@hotmail.com; 3 Psychiatry Department, Erenköy Training and Research Hospital for Mental and Neurological Diseases, University of Health Sciences, Istanbul 34736, Turkey; mine.ergelen@gmail.com (M.E.); drbeyzasarioz@gmail.com (B.A.); drpaltun@gmail.com (S.C.P.); 4 Psychology Department, Faculty of Humanities and Social Sciences, Istanbul Ticaret University, Istanbul 34445, Turkey; bilicirabia@gmail.com

**Keywords:** smoking cessation, tobacco use disorder, nicotine addiction, cognitive distortion, misperception, seeking treatment, treatment utilization

## Abstract

**Background/Objectives:** Despite the availability of effective pharmacotherapy and evidence-based treatments, a substantial proportion of smokers do not seek treatment. This study aims to explore the cognitive distortions associated with not seeking evidence-based smoking cessation treatment and to identify cognitive barriers. **Methods:** The research conducted in Istanbul between October and December 2017 employs a cross-sectional design and includes two groups: a treatment-seeking group comprising 156 patients diagnosed with tobacco use disorder and a non-treatment seeking group of 78 patients with tobacco use disorder who had never sought professional help for smoking cessation. A comprehensive data collection process was used, including sociodemographic information, cognitive distortion assessment using the cognitive distortions scale, a smoking-related cognitive distortions interview and the Fagerström Test for Nicotine Dependence. **Results:** While no significant sociodemographic differences were observed between the treatment-seeking and non-treatment-seeking groups, the study found that higher nicotine dependence was associated with a higher likelihood of seeking treatment. The treatment-seeking group displayed significantly higher levels of “all-or-nothing thinking” cognitive distortions related to smoking and smoking cessation. Conversely, the non-treatment-seeking group exhibited elevated levels of cognitive distortions such as “labeling”, “mental filtering”, “should statements” and “minimizing the positive” regarding receiving smoking cessation treatment. **Conclusions:** Understanding the cognitive distortions associated with treatment-seeking behavior for tobacco use disorder is crucial for developing targeted public-based interventions, public service announcements for tobacco use prevention and encouraging individuals to seek evidence-based treatment. Addressing these cognitive distortions can also potentially enhance the effectiveness of smoking cessation programs and reduce the global burden of tobacco-related diseases and mortality.

## 1. Introduction

Tobacco use is a paramount public health issue, ranking among the leading causes of preventable diseases and fatalities globally. The term “smoker” is used to describe an adult who has smoked 100 cigarettes in their lifetime and who currently smokes cigarettes. Beginning in 1991, this group was divided into two categories: “everyday” smokers (previously called regular smokers) and “somedays” smokers (previously called occasional smokers) [1]. According to the World Health Organization (WHO), approximately 1.3 billion people are smokers worldwide, with an estimated seven million deaths attributed to smoking annually [2]. Alarmingly, it is projected that the number of individuals succumbing to smoking-related causes will surge to 8.4 million by 2030 [3].

Pharmacotherapy has demonstrated its efficacy in smoking cessation treatment, while other methods remain unconvincing in the absence of substantial evidence, except for supportive therapy that incorporates motivational interviewing and cognitive behavioral approaches. Data from clinical trials have shown that the combination of pharmacotherapy and behavioral counselling is more effective in smoking cessation than the use of either alone [4,5]. Astonishingly, despite over two-thirds of adult smokers expressing a desire to quit, and around 40% making earnest attempts each year, merely 20–30% of them resort to evidence-based treatment methods like pharmacological intervention or behavioral counseling [6,7]. It has also been reported that the one-year quit rate for patients who tried to quit smoking with minimal support, such as self-help materials, brief advice from general practitioners or proactive telephone counselling was 1–2%. Conversely, the abstinence rate with optimal medication and support therapy was around 25% [6,8,9].

Previous research on tobacco use has primarily focused on enhancing the effectiveness of existing treatments or devising novel approaches [10,11]. A predominant focus of these studies has been the determinants of smoking cessation, with an overwhelming emphasis on the significance of seeking medical assistance and treatment. It is noteworthy that the populations with the highest prevalence of smoking exhibit the lowest treatment utilization rates [8,12]. In Turkey, the overall smoking prevalence was found to be 30.5% for the entire group, 15.7% for women and 46.1% for men, when occasional smokers were included. Turkey has one of the highest smoking prevalences, and the research on smoking cessation has primarily revolved around the analysis of the sociodemographic data of patients attending smoking cessation clinics, mirroring the patterns observed in other countries [13,14,15,16,17]. Studies that delve into the factors influencing the use of smoking cessation treatments usually juxtapose the sociodemographic characteristics of those who seek treatment against those who do not among individuals attempting to quit [18,19]. However, a limited number of studies have explored cognitive barriers in seeking smoking cessation treatment and abnormal cognitive and motivational processes that perpetuate smoking dependence, particularly in the pretreatment period [20,21]. Consequently, there is relatively little data on the cognitive and behavioral aspects of individuals trying to quit smoking [19,22].

In the light of the available literature on tobacco use disorder (TUD), it is evident that the engagement of medical support or treatment during smoking cessation attempts substantially enhances abstinence rates. However, the paradox lies in the fact that most smokers do not actively seek treatment when embarking on a cessation attempt [19]. This might be attributed to the inadequate alignment of treatment providers with the expectations and needs of smokers [23]. Factors such as a lack of awareness regarding treatment services, insufficient outreach efforts by healthcare services and insurers, and the absence of personalized interventions, particularly in the initial stages of cessation, contribute to the non-treatment seeking behavior among those seeking to quit smoking [19,21,24].

Cognitive distortions, irrational or exaggerated thoughts predisposing individuals to emotional and behavioral challenges, have been linked not only to health risks but also to the perception of susceptibility to illnesses and health-risk behaviors such as smoking. The assessment of generalized cognitive distortions has been employed to identify individuals with potential health risks and facilitate targeted interventions [25]. Cognitive distortions associated with addictive behaviors, including predictive control, catastrophizing, personalization and selective abstraction, have been a focal point of research [25,26,27,28]. However, cognitive distortions specifically linked to the non-utilization of smoking cessation treatment during cessation attempts have not received substantial attention.

In this context, understanding the cognitive distortions associated with seeking treatment for smoking cessation has the potential to enhance the utilization rates of evidence-based treatment services, ultimately leading to improved smoking cessation rates. This study aims to investigate the cognitive distortions that could impede smokers from utilizing evidence-based smoking cessation treatments.

## 2. Material and Methods

### 2.1. Sample

The study adopted a cross-sectional design and focused on patients who consecutively attended the smoking cessation outpatient unit of Erenköy Training and Research Hospital for Psychiatry and Neurology in Istanbul during a three-month period, between 1 October and 31 December 2017. Exclusion criteria included individuals with cognitive deficits, psychotic or mood disorder symptoms, dementia, intellectual disability or severe physical illness. One hundred and sixty-eight patients diagnosed with tobacco use disorder (TUD) during a clinical interview according to DSM-5 criteria were included in the treatment-seeking (TS) group at the beginning of the treatment. Additionally, twelve patients with low or low-to-moderate scores (less than 5 points) on the Fagerström Test for Nicotine Dependence were excluded. This exclusion aimed to account for the association between lower nicotine dependence and reduced treatment seeking and utilization as indicated by previous research [29,30]. Ultimately, a total of 156 patients were included in the TS group.

The non-treatment-seeking (non-TS) group comprised seventy-eight patients who visited Primary Healthcare Centers in the same neighborhood between 1 January and 28 February 2018. These patients were diagnosed with TUD according to DSM-5 criteria and had moderate to higher levels of nicotine dependence as identified by the Fagerström Test for Nicotine Dependence. Additionally, they had made attempts to quit smoking in the past year but had never sought medical help for smoking cessation.

### 2.2. Data Collection Instruments

*Sociodemographic and Clinical Data Form*: Researchers developed this form to gather information about participants’ age, gender, employment status, marital status, income and education levels, employment status, smoking duration, average daily cigarette consumption, frequency of cessation attempts and the longest duration of smoking cessation.

*Cognitive Distortions Scale* (CDS): This scale, developed by Covin et al. [31], assesses cognitive distortions using a 7-point scale, with 20 items evaluating 10 cognitive dimensions. These dimensions include mind-reading, catastrophizing, all-or-nothing thinking, emotional reasoning, labeling, mental filtering, overgeneralization, personalization, should statements and minimizing the positive. CDS includes ratings in two domains: interpersonal and personal achievement. The scale was adapted into Turkish and validated in 2014 [32].

*Smoking-Related Cognitive Distortions Interview:* To assess dysfunctional thoughts related to tobacco use and smoking cessation, a structured interview was developed by the researchers, focusing on cognitive characteristics derived from the CDS. The interview aimed to evaluate the most frequently used cognitive distortions among TUD patients regarding smoking. The questions of the interview were reviewed and validated by three experienced cognitive behavioral therapy (CBT) therapists after conducting interviews with 90 outpatients consecutively admitted to the smoking cessation unit over one month. The interview aimed to assess cognitive distortions in nine different cognitive dimensions in two headings: smoking–smoking cessation as first heading and receiving smoking cessation treatment as second heading. Upon the assessment of the cognitive distortions associated with smoking in the interviews conducted to determine the adapted questions, the results indicated that only a small proportion of patients employed the use of mind reading, whereas other distortion items were frequently utilized by the patients. Consequently, the authors elected to exclude the mind reading item from the adaptation of the cognitive distortions scale. The interview, lasting around 45–60 min, was conducted by the same researcher. Participants rated items containing cognitive distortions related to smoking and receiving smoking cessation treatment on a Likert scale ranging from 1 to 10.

*Fagerström Test for Nicotine Dependence (FTND):* Developed by Fagerström and Schneider in 1989 [33], this test assesses the level of nicotine dependence. Scores range from 0 to 11, with lower scores indicating lower dependence and higher scores indicating higher dependence. The test has been translated into Turkish, and its validity and reliability were investigated [34].

### 2.3. Statistical Analysis

Statistical analyses were conducted using SPSS version 16. Normality of the variables was assessed visually using histograms and probability graphs, as well as analytically through Kolmogorov–Smirnov/Shapiro–Wilk tests. The chi-squared test was employed for two-group comparisons of categorical variables. Mann–Whitney U test was used for the comparison of non-parametric nominal and ordinal variables. A significance level of *p* < 0.05 was considered statistically significant. A post hoc power calculation was conducted to determine the adequacy of the sample size at a 95% confidence level using the “G. Power-3.1.9.4” program. The analysis determined that, at an α = 0.05 level, a sample size of 156 individuals seeking treatment and 78 individuals not seeking treatment has adequate power (Power (1-β error probability) = 0.999).

## 3. Results

### 3.1. Sociodemographic Characteristics

A total of 234 individuals participated in the study, with gender distribution as follows: in the TS group, there were 76 females (48.7%) and 80 males (51.3%), while in the non-TS group, there were 47 females (60.3%) and 31 males (39.7%). The average age of the participants was 44.01 ± 11.50 in the non-TS group and 39.69 ± 11.20 in the TS group. Detailed sociodemographic characteristics of the participants are presented in Table 1.

### 3.2. Cigarette Use Related Clinical Characteristics

An analysis of the patients’ smoking duration in years revealed no statistically significant difference between the TS group (20.72 ± 10.59) and the non-TS group (22.99 ± 12.35) (*p* = 0.183). The average number of cigarettes smoked per day was statistically similar between the two groups (*p* = 0.161). When examining the frequency of attempts to quit smoking, it was observed that the TS group had a significantly lower frequency (2.04 ± 1.85) compared to the non-TS group (2.56 ± 2.27) (*p* = 0.044). Furthermore, there was no statistically significant difference between the two groups in terms of the longest cessation duration (*p* = 0.244). Table 2 provides an overview of the cigarette-use-related clinical characteristics of the participants.

### 3.3. Assessment of the Participants’ General and Smoking-Related Cognitive Distortions

The general cognitive distortions (CDs) of the patients were analyzed using the CDS (presented in Table 3). The assessment of smoking-cessation-related CDs (as shown in Table 4) and receiving-smoking-cessation-treatment-related CDs (as shown in Table 5) was carried out through a smoking-related CD assessment.

Upon examining the frequency of using general CDs in both groups, it was observed that “mindreading” scores were significantly higher in the TS group in both interpersonal (*p* = 0.003) and personal achievement domains (*p* = 0.011). Additionally, “minimizing the positive” scores were also significantly higher in the TS group in both the interpersonal (*p* = 0.023) and personal achievement domains (*p* = 0.014).

In terms of smoking–smoking-cessation-related CD scores, there was no significant difference between the two groups except for the “all-or-nothing thinking” CD scores, which were significantly higher in the TS group (*p* = 0.009).

Regarding receiving-smoking-cessation-treatment-related CDs, the “labeling” (*p* = 0.037), “mental filtering” (*p* = 0.034), “should statements” (*p* = 0.008) and “minimizing the positive” (*p* = 0.027) CD scores were significantly higher in the non-TS group compared to the TS group.

## 4. Discussion

The comparison of the TS and non-TS groups revealed several insights into the sociodemographic and clinical characteristics of individuals seeking treatment for smoking cessation.

Promoting smoking cessation initiatives targeted at women is an important public health imperative. Although the prevalence of cigarette smoking is slightly lower among women than among men, women are reported to be more likely to suffer from the adverse health effects associated with smoking. In addition, women may face greater challenges in utilizing treatment than men due to specific barriers including concerns about smoking-related weight change and effects on the menstrual cycle [35]. However, studies examining gender differences in treatment utilization have reported conflicting results. Data collected from 11,536 households in Turkey in 2012 revealed that almost half of smokers (46%) had tried to quit smoking in the past 12 months, with a higher rate of cessation attempts among women (48.8%) than men (45.1%). Women were also found to be more prone to utilizing pharmacological treatments and consulting methods for quitting than men (14.5% vs. 13.3% and 9.5% vs. 7.5%, respectively) [36]. A study conducted in the United States which included 12,207 participants who had attempted to quit smoking in the past year revealed that females were more likely than males to receive treatment during their quitting attempt. The rate of using behavioral treatment alone or in combination with medication was also significantly higher among female patients [30]. However, Allen et al. [35] reported a slightly lower utilization of smoking cessation services among women. Our results have revealed no statistically significant difference between the two groups in terms of gender (*p*: 0.096). Similar to the latter report, in our study, there were slightly more men than women in the TS group. However, the ratio of men/women in the TS group was close to each other while the number of women was higher than that of men in the non-TS group. This could result from the fact that most of the patients who visit primary healthcare centers are women in Turkey [37].

According to the Global Adult Tobacco Survey (GATS) report, the 25–44 age group had the highest values for both cigarette use (51.9%) and manufactured cigarette use (50.0%) when the distribution of tobacco use by age was examined. Despite being the most comprehensive study on cigarette use in Turkey, it does not consider the effects of age on getting help while quitting smoking [36]. A survey conducted in our country revealed that the mean age of individuals attending a smoking cessation clinic was 38.4 years ± 12.5, based on a sample of 2119 people [38]. Another study from Turkey, in which 320 patients who referred to a smoking cessation clinic were evaluated, indicated that the average age was 42.6 ± 13.5 [39]. Our findings regarding age were consistent with the studies conducted in Turkey and other countries.

### 4.1. Comparison of the Groups in Terms of Sociodemographic, Clinical and Cigarette Use Characteristics

In the present study, there was no significant difference between the TS and non-TS groups in terms of marital status, income level, employment status and education level (*p* values were 0.135, 0.372, 0.773 and 0.636, respectively). These results imply that other factors, aside from sociodemographic disparities, may be hindering individuals from seeking treatment, which is in line with the aim of the study to investigate cognitive distortions related to seeking smoking cessation treatment.

Previous studies have focused on identifying the factors that influence treatment seeking by examining the characteristics of individuals who seek treatment. Hughes et al. [19] reported that the age, gender, educational status, time of smoking the first cigarette of the day, number of cigarettes smoked a day, longest cessation duration, physical illness, or reason for cessation and being a woman were predictors of receiving smoking cessation treatment. Additionally, being older, smoking more cigarettes in a day and having seen a healthcare professional within the past year were found to be predictors of using pharmacotherapy.

Comparing the cigarette use characteristics revealed no statistically significant difference between the groups (e.g., duration of smoking, number of cigarettes per day). Data show that individuals who have been smoking for more than 25 years have the highest rate of seeking treatment [30]. Research suggests that the frequency of smoking cigarettes per day may have an association with treatment utilization [19]. When examining the number of cigarettes smoked per day, the TS group was found to have a higher number of cigarettes (25.03 ± 11.5) compared to the non-TS group (22.33 ± 8.41). This difference, however, was not statistically significant. (*p*: 0.161). In our research, the nicotine addiction level of the TS group (6.48 ± 1.88) was significantly higher than the non-TS group (5.90 ± 1.93; *p*: 0.016). Considering that both groups comprised patients with moderate, high, and very high levels of tobacco use, this finding corroborates the existing literature indicating that patients with a higher degree of addiction are more likely to seek treatment [19,30,40]. When examining the number of attempts to quit smoking, it was observed that the non-TS group had a significantly higher rate of cessation attempts compared to the TS group (*p* = 0.044). This suggests that the non-TS group may have attempted to quit smoking more frequently on their own without medical support.

### 4.2. Evaluation of the Effects of General and Smoking-Specific CDs on Seeking Treatment for Smoking Cessation

Studies have shown that people who are trying to quit smoking often do not seek medical help, and it is important to identify the personal barriers that are preventing them from seeking treatment. In addition, to develop effective treatments for TUD, it seems essential to identify the factors that can be modified to encourage people to seek and receive treatment [19,24].

According to cognitive behavioral theory, behaviors and thoughts have reciprocal effects and may influence each other. Understanding cognitive distortions, which are maladaptive cognitive schemas altering an individual’s perceptions of self, others or the future in the context of perceived disease risk and susceptibility, has been instrumental in predicting medical adherence and health behaviors in people with a variety of mental health and medical conditions including major depressive disorder and diabetes mellitus [41,42]. This perspective is crucial in interpreting smoking-specific CDs, as it may contribute to changing individuals’ smoking-cessation-treatment-seeking behavior. 

In the present study, when comparing the cognitive distortion scale scores of the two groups, it was found that only “all-or-nothing thinking” related to smoking and smoking cessation was significantly higher in the TS group. Addictive thinking is often characterized by cognitive distortions such as all-or-nothing thinking, disqualifying the positives, catastrophizing, and labeling. When individuals adopt an all-or-nothing, dichotomous perspective on recovery, they often experience heightened feelings of overwhelm and are more inclined to avoid long-term processes and goals such as treatment plans in favor of seeking immediate relief [43]. However, interestingly, our results on cognitive distortions related to smoking and smoking cessation were contradictory with the literature. Although this way of thinking makes it difficult to start and maintain a treatment program, it may also have led individuals to seek medical help for this difficult goal with the thought that “I cannot quit smoking without treatment”. In individuals who did not seek treatment, it is thought that they may not have sought medical help because they thought “that they could quit smoking on their own”. In this context, although there was no statistically significant difference between the two groups in terms of duration of smoking, the number of smoking cessation attempts was significantly higher in the non-TS group; the authors concluded that this finding could also be related to the smoking- and smoking-cessation-related CDs.

Other cognitive distortions related to smoking and smoking cessation were similar between the two groups in all other cognitive dimensions; suggesting that smokers share similar CDs related to smoking and smoking cessation to similar degrees regardless of whether they seek treatment, and these are related to other factors that prevent them from seeking help.

Regarding receiving-smoking-cessation-treatment-related CDs, our study revealed significant findings: labeling, mental filtering, should statements and minimizing the positive levels were statistically higher in the non-TS group.

In the non-TS group, the most frequent cognitive distortion (CD) was “treatment is for patients, I’m not sick, so I don’t need to visit a doctor”, which is a labeling-style cognitive distortion. As smoking is not considered as likely to cause psychosocial problems (e.g., work, legal, social, family) as alcohol or drug use disorders, is not commonly considered as an addictive disorder by the smokers [44]. Our results suggest that instead of asking “do you smoke”? during medical check-ups, questioning patients if they have “tobacco use disorder” could help the patient to recognize smoking as an addictive disease and encourage them to receive medical help for their addiction. The authors also suggest that community-based interventions and public service announcements consider the importance of emphasizing TUD as a medical disease as well as smoking.

It is possible to interview patients in order to ascertain their views on the advantages and disadvantages of the cognition like “it should not have any side effects for me to use drugs”, which is a “should statement” seen more common in the non-TS group. With a profit and loss analysis conducted by comparing the risks of continuing to smoke and the disadvantages of medications, the patient may develop a more flexible attitude and participate in treatment. Studies have revealed that informing patients about the drugs and their likely side effects may result in better compliance to the medications, both in physical illness and addiction treatment [45,46].

Regarding the receiving-smoking-cessation-treatment-related CDs, the non-TS group was also characterized by mental filtering and minimizing the positive. For example, they thought that the treatment efficacy was only 30%, and “if their friend quit smoking with medications, it was only due to luck”. These CDs can be intervened with during an interview with Socratic questioning such as “how many times is 30% when compared with 3%”? The results of the study suggest that focusing on cognitions related to smoking cessation treatment and the internal barriers that prevent individuals from seeking treatment, both in face-to-face interviews and in community-based interventions such as public service announcements, could make a significant contribution to treatment-seeking behavior.

Although there are numerous tobacco treatment training programs globally, there is a paucity of information concerning the profile of individuals who seek advanced training in TUD treatment. The management of TUD has become increasingly intricate, necessitating the customization of therapeutic strategies for individual needs to encompass the spectrum of tobacco products [47]. Another crucial aspect of smoking cessation is the implementation of educational and prevention programs, particularly for adolescents. Despite a clear association between e-cigarette usage and unfavorable health outcomes, adolescents hold erroneous perceptions about these products, which contribute to both the initiation and continued utilization of e-cigarettes. As novel forms of tobacco products enter the market and become more prevalent among youth, there is a pressing need for educators tasked with tobacco prevention education to possess the knowledge and expertise to address and dispel the misperceptions held by this demographic [48]. Accordingly, the authors propose that the findings of the present study may benefit the training of healthcare providers and community educators and contribute to public education and prevention programs for both adults and adolescents.

### 4.3. Limitations of the Study

Firstly, the present study is a cross-sectional study with a relatively small sample size. The study was conducted with a sample referring to the smoking cessation clinic of a hospital and the primary health care centers in the same neighborhood. In addition, the voluntary nature of the study could have introduced selection bias, leading to a non-representative sample, thus limiting the generalizability of the results. As the patients admitting to the primary health care centers are a fixed population connected to the physician, it is possible that the anonymity of the respondents may have potentially caused response bias, despite the questionnaires being designed to protect this anonymity. Finally, the authors note that the non-treatment-seeking group was recruited from primary care centers, while the treatment-seeking group came from a smoking cessation clinic. The different recruitment settings could potentially lead to confounding. Thus, the authors cannot make strong causal inferences about the relationship between cognitive distortions and treatment-seeking behavior based on this study.

## 5. Conclusions

The results highlight an important domain in the research for smoking cessation treatment. The present study revealed that smokers’ cognitions and behaviors regarding seeking treatment may vary. It could be assumed that individual and community-based interventions to change CDs about the importance of seeking professional help to quit smoking may change treatment utilization rates. Accordingly, focusing on cognitive distortions such as “labelling, mental filtering, should statements and minimizing the positive” in smoking cessation clinics and primary healthcare centers may provide a significant contribution to treatment seeking and utilization and also the findings of the study may benefit the training of healthcare providers and community educators for the education and prevention programs supporting smoking cessation. Further studies with larger samples will provide more knowledge about cognitive distortions related to seeking smoking cessation treatment.

## Figures and Tables

**Table 1:** Sociodemographic characteristics of the groups.

		Non-Treatment-Seeking Group *n*, (%)	Treatment-Seeking Group *n*, (%)	U/Z * Chi-Squared/df	*p*
Gender	Male	31 (39.7%)	80 (51.3%)	2.777/ 1	0.096
	Female	47 (60.3%)	76 (48.7%)		
Age ± SD		44.01 ± 11.50 (44.00)	39.69 ± 11.20 (37.00)	4761.5/ −2710 *	0.007
Marital status	Married	50 64.1%	84 53.8%	2.235/ 1	0.135
	Single	28 35.9%	72 46.2%		
Income Level	Low	10 12.8%	26 16.7%	5791.0/ −0.892 *	0.372
	Moderate	65 83.3%	126 80.8%		
	High	3 3.8%	4 2.6%		
Employment	Yes	49 62.8%	101 64.7%	0.149/ 1	0.773
	No	29 37.2%	55 35.3%		
Level of education completed (years) successfully ± SD		11.22 ± 4.75 (11.00)	11.58 ± 4.35 (12.00)	5854.5/ −0.473	0.636

* indicates Z values.

**Table 2:** Smoking characteristics of the groups.

		Non-Treatment-Seeking Group Avg ± SD	Treatment-Seeking Group Avg ± SD	U/Z	*p*
Duration of smoking (year)		22.99 ± 12.35	20.72 ± 10.59	5435.0/ −1.332	0.183
Average number of cigarettes smoked per day		22.33 ± 8.41	25.03 ± 11.50	5410.0/ −1.400	0.161
Number of cessations		2.56 ± 2.27	2.04 ± 1.85	5125.0/ −2.014	0.044
Longest cessation duration	Up to 1 week	34	72	5527.5/ −1.165	0.244
		43.6%	46.2%		
	Between 1 week and 1 month	16	25		
		20.5%	16.0%		
	Between 1 month and 3 months	9	19		
		11.5%	12.2%		
	Between 3 month and 6 months	8	11		
		10.3%	7.1%		
	Between 6 months and 1 year	3	14		
		3.8%	9.0%		
	Longer than 1 year	8	15		
		10.3%	9.6%		

**Table 3:** Cognitive distortion scale scores of the groups (with friends, spouse, or family).

	Non-Treatment-Seeking Group Avg ± SD		Treatment-Seeking Group Avg ± SD		U/Z	*p* */*p* **
Mindreading: IP/PA	3.99 ± 1.78	3.96 ± 1.78	4.72 ± 1.74	4.60 ± 1.83	4668.5/−2.942 4853.5/−2.554	0.003 */ 0.011 **
Catastrophizing: IP/PA	3.58 ± 1.86	3.81 ± 1.69	3.47 ± 1.75	3.46 ± 1.81	5966.5/−0.245 5316.5/−1.594	0.807 */ 0.111 **
All-or-nothing thinking: Interpersonal domain IP/PA	3.46 ± 1.76	3.50 ± 1.80	3.44 ± 1.73	3.62 ± 1.76	6079.0/−0.010 5785.5/−0.621	0.992 */ 0.535 **
Emotional reasoning: IP/PA	3.44 ± 1.49	3.29 ± 1.85	3.70 ± 1.93	3.36 ± 1.89	5676.0/−0.848 5978.0/−0.220	0.397 */ 0.826 **
Labeling: IP/PA	2.79 ± 1.84	2.83 ± 1.75	3.10 ± 1.88	3.08 ± 1.85	5491.5/−1.244 5634.5/−0.940	0.213 */ 0.347 **
Mental filtering: IP/PA	3.09 ± 1.43	3.12 ± 1.58	3.37 ± 1.83	3.36 ± 1.83	5672.5/−0.857 5717.0/−0.763	0.392 */ 0.445 **
Overgeneralization: IP/PA	3.10 ± 1.79	2.78 ± 1.71	3.59 ± 2.07	3.23 ± 1.99	5327.0/−1.573 5384.5/−1.463	0.116 */ 0.143 **
Personalization: IP/PA	3.12 ± 1.75	3.01 ± 1.48	3.28 ± 1.74	3.13 ± 1.63	5721.0/−0.755 5897.0/−0.390	0.450 */ 0.696 **
Should statements: IP/PA	3.14 ± 1.77	2.79 ± 1.69	3.26 ± 1.88	3.34 ± 1.87	5916/−0.349 5065/−2.122	0.727 */ 0.034 **
Minimizing the positive: IP/PA	2.31 ± 1.48	2.26 ± 1.45	2.90 ± 1.82	2.89 ± 1.81	5003.0/−2.279 4918.5/−2.457	0.023 */ 0.014 **

IP: Interpersonal Domain; PA: Personal Achievement Domain. *p* *: significance between IP scores; *p* **: significance between AP scores.

**Table 4:** Smoking-cessation-related cognitive distortion levels of the groups.

	Non-Treatment-Seeking Group Avg ± SD	Treatment-Seeking Group Avg ± SD	U/Z	*p*
Catastrophizing/Smoking–smoking cessation	6.49 ± 2.86	5.85 ± 2.93	5183.5/ −1.215	0.061
All-or-nothing thinking/Smoking–smoking cessation	7.98 ± 2.82	6.74 ± 3.55	4863.5/ −2.596	0.009
Emotional reasoning/Smoking–smoking cessation	2.67 ± 3.24	2.61 ± 3.23	5953.5/ −0.114	0.909
Labeling/Smoking–smoking cessation	4.88 ± 3.45	4.08 ± 3.52	5156.5/ −1.707	0.088
Mental filtering/Smoking–smoking cessation	2.92 ± 3.28	3.43 ± 3.54	5611.0/ −1.007	0.314
Overgeneralization/Smoking–smoking cessation	2.26 ± 3.35	2.37 ± 3.33	5900.0/ −0.417	0.677
Personalization/Smoking–smoking cessation	6.68 ± 3.48	6.61 ± 3.28	5953.0/ −0.275	0.783
Should statements/Smoking–smoking cessation	5.62 ± 3.47	5.82 ± 3.59	5828.0/ −0.454	0.650
Minimizing the positive/Smoking–smoking cessation	2.33 ± 3.31	2.18 ± 3.10	5910.0/ −0.388.	0.698

**Table 5:** Receiving-smoking-cessation-treatment-related cognitive distortion levels of the groups.

	Non-Treatment-Seeking Group Avg ± SD	Treatment-Seeking Group Avg ± SD	U/Z	*p*
Catastrophizing/Receiving treatment—help in smoking cessation	4.33 ± 3.75	4.97 ± 3.67	5498.5/ −1.215	0.224
All-or-nothing thinking/Receiving treatment—help in smoking cessation	6.05 ± 3.55	6.56 ± 3.30	5625.0/ −0.960	0.337
Emotional reasoning/Receiving treatment—help in smoking cessation	4.10 ± 3.66	4.21 ± 3.35	5919.5/ −0.263	0.793
Labeling/Receiving treatment—help in smoking cessation	1.83 ± 3.17	0.84 ± 1.94	5261.0/ −2.084	0.037
Mental filtering/Receiving treatment—help in smoking cessation	3.31 ± 3.08	2.43 ± 2.79	5093.0/ −2.115	0.034
Overgeneralization/Receiving treatment—help in smoking cessation	1.63 ± 2.69	1.56 ± 2.57	6030.5/ −0.126	0.900
Personalization/Receiving treatment—help in smoking cessation	2.46 ± 3.49	2.24 ± 3.49	5652.5/ 0.982	0.326
Should statements/Receiving treatment—help in smoking cessation	5.79 ± 3.86	4.47 ± 3.57	4807.0/ −2.658	0.008
Minimizing the positive/Receiving treatment—help in smoking cessation	2.46 ± 3.19	1.59 ± 2.50	5095.5/ −2.219	0.027

## Data Availability

The datasets used and analyzed during the current study are available from the corresponding author upon reasonable request.
